# Neurocognitive changes after awake surgery in glioma patients: a retrospective cohort study

**DOI:** 10.1007/s11060-019-03341-6

**Published:** 2019-12-04

**Authors:** Emma van Kessel, Tom J. Snijders, Anniek E. Baumfalk, Carla Ruis, Kirsten M. van Baarsen, Marike L. Broekman, Martine J. E. van Zandvoort, Pierre A. Robe

**Affiliations:** 1 Department of Neurology & Neurosurgery, University Medical Center Utrecht/UMC Utrecht Brain Center, G03.232, PO Box 85500, 3508 XC Utrecht, The Netherlands; 2 Helmhotz Institute, Utrecht University, Room 1715, Heidelberglaan 1, 3584 CS Utrecht, The Netherlands; 3 Department of Neurosurgery, Leiden University Medical Center, PO Box 9600, 2300 RC Leiden, The Netherlands

**Keywords:** Neurocognitive functioning changes, Glioma, Neuropsychology, Brain tumor, Determinants of neurocognitive functioning

## Abstract

**Purpose:**

Deficits in neurocognitive functioning (NCF) frequently occur in glioma patients. Both treatment and the tumor itself contribute to these deficits. In order to minimize the harmful effects of surgery, an increasing number of patients undergo awake craniotomy. To investigate whether we can indeed preserve cognitive functioning after state-of-the art awake surgery and to identify factors determining postoperative NCF, we performed a retrospective cohort study.

**Methods:**

In diffuse glioma (WHO grade 2–4) patients undergoing awake craniotomy, we studied neurocognitive functioning both pre-operatively and 3–6 months postoperatively. Evaluation covered five neurocognitive domains. We performed analysis of data on group and individual level and evaluated the value of patient-, tumor- and treatment-related factors for predicting change in NCF, using linear and logistic regression analysis.

**Results:**

We included 168 consecutive patients. Mean NCF-scores of psychomotor speed and visuospatial functioning significantly deteriorated after surgery. The percentage of serious neurocognitive impairments (− 2 standard deviations) increased significantly for psychomotor speed only. Tumor involvement in the left thalamus predicted a postoperative decline in NCF for the domains overall-NCF, executive functioning and psychomotor speed. An IDH-wildtype status predicted decline for overall-NCF and executive functioning.

**Conclusions:**

In all cognitive domains, except for psychomotor speed, cognitive functioning can be preserved after awake surgery. The domain of psychomotor speed seems to be most vulnerable to the effects of surgery and early postoperative therapies. Cognitive performance after glioma surgery is associated with a combination of structural and biomolecular effects from the tumor, including IDH-status and left thalamic involvement.

**Electronic supplementary material:**

The online version of this article (10.1007/s11060-019-03341-6) contains supplementary material, which is available to authorized users.

## Introduction

Diffuse gliomas are progressive primary brain tumors that are almost invariably fatal, despite recent advances in treatment. However, since these advances have led to improved life expectancy, researchers and clinicians are simultaneously focusing efforts on maintaining quality of life. One of the major determinants of quality of life in glioma patients is neurocognitive functioning (NCF) [1]. Cognitive deficits occur in a substantial proportion of glioma patients even before any antineoplastic treatment is given, and can occur or worsen as a complication of treatment itself [2].

Awake glioma surgery with intra-operative testing of NCF aims to reduce the risk of such complications. Since functional brain anatomy and extent of tumor invasion differ between individual patients, awake surgery offers the opportunity to maximize tissue resection while sparing cognitive and other neurological functions. In the initial development of awake glioma surgery, the main focus was on language, sensorimotor functions and vision [3, 4]. In recent years, focus is shifting toward other cognitive domains such as executive functioning, psychomotor speed, and memory which are less dependent on one specific location and are more difficult to test intra-operatively [5]. These cognitive domains are found to be frequently impaired in treatment-naive glioma patients and may also be vulnerable to the effects of surgery and adjuvant treatments [6, 7].

Both patient-, treatment-, and tumor-related factors likely play an important role in cognitive changes after surgery. Unraveling the factors that influence cognitive changes after therapy could facilitate the development of new, personalized treatment strategies to maintain cognitive functioning at the best attainable level. It is, however, not yet possible to accurately predict neurocognitive changes after surgery in an individual patient [8–10]. A first step in this path is to quantify neurocognitive changes after therapy across the different cognitive domains, in sufficiently large series of patients. The second step is to correlate preoperative characteristics with these outcomes.

We performed a retrospective study to evaluate NCF in glioma patients before and after surgery and—where applicable—initial adjuvant therapy. The aim of this study is to give an overview of changes in NCF in glioma patients after treatment and to study which factors influence these cognitive changes. We hypothesize that (a) NCF is better preserved in domains that are dependent on specific locations (language, visuospatial functioning) than in domains that are dependent on more widespread cerebral networks (executive functioning, speed and memory), and consequently more difficult to test intraoperatively, and (b) that a combination of patient- and tumor- related factors determine the postoperative neurocognitive outcome [11, 12].

## Methods

### Design

We performed a single-center retrospective study, in a consecutive cohort of diffuse glioma patients who underwent neuropsychological testing as part of their routine pre-operative work-up and of the post-operative evaluation for awake brain surgery between 2010 and 2016. Neuropsychological testing took place between 2010 and April 2017.

In the study sample, we studied overall NCF as well as domain-specific NCF for five neurocognitive domains. Data are reported according to STROBE-criteria (Online Resource 1)). Each neuropsychological test was scored according to standardized scoring criteria. The uncorrected scores were transformed into Z-scores based on the mean and standard deviation of the published norms for normative comparisons. All these neuropsychological data were prospectively collected in a database.

We also collected data about patient—and tumor characteristics (Online Resource Table 1) including data on tumor grade and molecular markers, which were converted into the WHO 2016 classification [13]. More information about the study design can be found in “Online Resource 2”.

### Analysis

We performed analyses of neuropsychological functioning data with SPSS (IBM SPSS Statistics, 25.0.0), on two levels of outcome:

1. Group-level: difference of the mean z-value of the study sample as a whole between pre- and post-operative assessment, per domain and for overall neurocognitive functioning. We performed a paired *T* test to evaluate whether a significant change of mean domain-specific Z-scores occurred. We performed a Wilcoxon related-sample test instead, if data was not normally distributed.
2. Individual patient-level:
  - by means of percentage of patients with test performance below the threshold of impairment (− 2 standard deviations (SD)) before and after surgery; this was calculated for each domain (“percentage impairment per domain”). To evaluate whether the proportion of patients with impairment differed significantly pre- and postoperatively, we performed a non-parametric McNemar-test that takes into account repeated measures.
  - difference of the NCF scores for each patient between pre and post-operative assessment per domain. We categorized the change in NCF into one of six categories: (1) change of − 2 SD or worse; (2) − 2 to − 1 SD; (3) − 1 to 0 SD; (4) 0 to + 1SD; (5) + 1 to + 2SD; (6) +2 SD or better.

We performed a subgroup analysis for patients who did not receive any treatment after surgery (for both levels of outcome), since changes in NCF scores in this group are not influenced by adjuvant post-operative therapies, and thus form the best representation of the effects of tumor surgery itself on NCF. We further performed subgroup analyses for low-grade glioma (LGG) patients and high-grade glioma (HGG) patients, again for both levels of outcome.

### Determinants of influence on changes in NCF

To study which determinants were of influence on cognitive changes we also performed analyses of data on two levels of outcome:

1. *A delta*-*Z*-*score* (continuous variable) for overall-NCF, as well as for five domains as described above.
2. The *delta*-*Z*-*score was dichotomized into cognitive decline* (decrease of Z-score of 1 or more) versus no or subtle cognitive decline (decrease of Z-score < 1, or increase), compared to the preoperative NCF.

We evaluated the predictive value of baseline characteristic (before surgery) on change in NCF, using univariable and multivariable linear and logistic regression analysis.

More details about the analyses are provided in the supplementary methods section (Online Resource 2) and in earlier published work (16).

## Results

### Clinical characteristics (Online Resource Table 1)

In total 270 patients underwent awake surgery between 2010 and 2016; 50 patients were excluded based on a diagnosis (non-glioma) or previous anti-tumor treatment; 52 were excluded because of insufficient neuropsychological data. In total 168 patients met our inclusion criteria at baseline and were included.

A total of 34 patients did not undergo post-operative neuropsychological assessment for several reasons (Online Resource Fig. 1), most commonly medical condition or patient refusal.

### Neurocognitive data

The number of patients with severe deterioration or large improvements was low for all different domains, most neurocognitive changes were subtle with a delta-Z-score between − 1 SD and + 1 SD. Online Resource Fig. 2 depicts the categorized results of the individual-level analyses (regarding changes in percentage of impairment per domain).

**Fig. 1:**
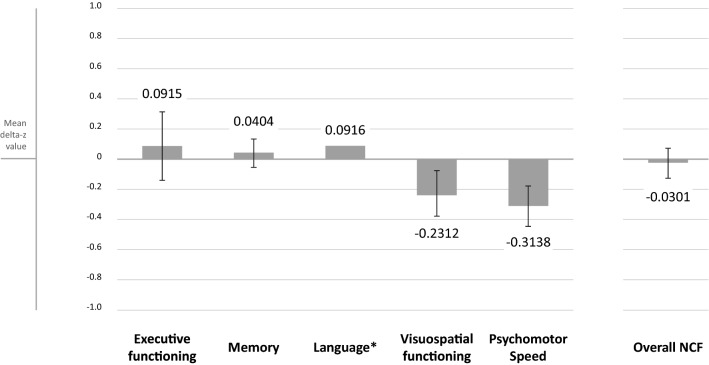
Group level analyses—post-operative change in mean cognitive scores (Z-scores) per domain. Asterisk: Wilcoxon related sample test performed, because these data was not normally distributed

Results of NCF analyzed at group level are shown in Fig. 1. Patients’ NCF scores slightly, but significantly decreased post-operatively for the domains of visuospatial functioning (mean difference 0.23, 95% confidence interval (CI) − 0.39 to − 0.08) and psychomotor speed (mean difference 0.31 (− 0.44 to − 0.18 95% CI)).

The subgroup analysis for patients (n = 50) who did not receive any postoperative treatment showed only significantly decreased mean post-operatively Z-scores for the domain visuospatial functioning [mean difference 0.29 (− 0.53 to − 0.06 95% CI)].

Subgroup analysis for HGG patients showed significant deterioration for the domain psychomotor speed [mean difference 0.45 (− 0.65 to − 0.24 95% CI)] and for LGG patients for the domains visuospatial functioning [mean difference 0.33 (− 0.54 to − 0.11 95% CI)] and psychomotor speed [mean difference 0.15 (− 0.28 to − 0.02 95% CI)].

Results for subgroup analyses are showed in Online Resource Fig. 3. Group level analyses with pre- and postoperative scores (Z-scores) per domain in boxplots are shown in Online Resource Fig. 4.

**Fig. 2:**
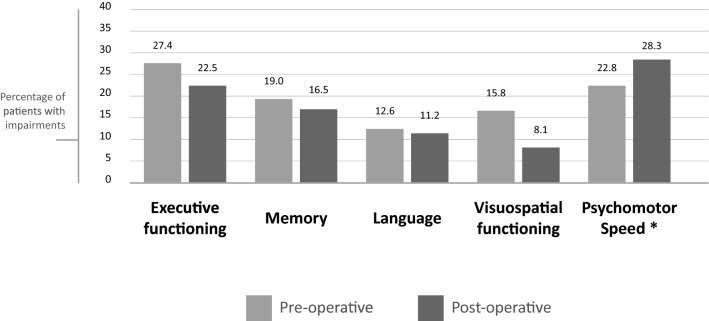
Individual level analyses—percentage of patients with domain-specific pre- and postoperative impairments

The difference between pre- and postoperative proportion of individuals with a serious cognitive impairment (-2SD) is shown in Fig. 2. These differences were only significant for psychomotor speed (preoperatively 22.8%, postoperatively 28.3%, p value = 0.008).

Subgroup analysis on individual level for patients who did not receive any postoperative treatment (which all were patients with LGG) showed comparable results to those who received post-operative treatment. HGG subgroup analyses did not show any significant postoperative changes. LGG analyses showed a significant increase in patients with an impairment for the domain speed (preoperatively 4.9%, postoperatively 14.8%, p value = 0.031). Results for subgroup analyses on individual level are shown in Online Resource Fig. 5.

### Determinants of changes in NCF (Fig. 3)

The results of the multivariable linear regression analyses are presented in Tables 1, 2, 3, 4, 5, and 6 (for overall-NCF and five cognitive domains). The results of the univariable analyses are shown in Online Resource 3.

**Fig. 3:**
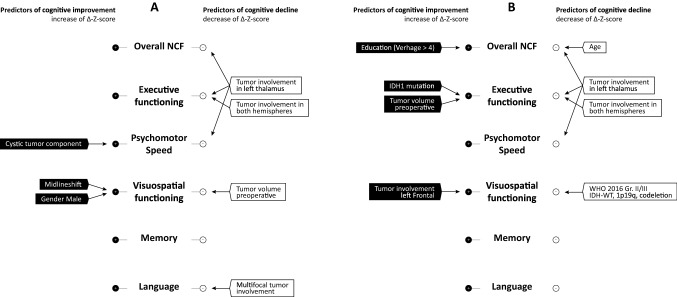
Summary of results of multivariable linear (**a**) and logistic (**b**) regression analyses. **a** Left side; significant determinants of cognitive improvement (positive mean delta Z-score), right side; determinants of cognitive decline (negative mean delta Z-score). **b** Left side; significant determinants of cognitive improvement, right side; significant determinants of cognitive decline (decrease of Z-score of 1 or more)

**Table 1:** Multivariable linear regression analyses for predicting delta-Z-scores (overall neurocognitive functioning)

Baseline variable	Univariable		Multivariable model 1		Multivariable model 2	
			R^2^ = 0.184 p = 0.006		R^2^ = 0.426 p = 0.012	
	B (95% CI)	p value	B (95% CI)	p value	B (95% CI)	p value
Age (at time of surgery)	− 0.009 (− 0.024 to 0.006)	0.249	− 0.002 (− 0.021 to 0.016)	0.814	0.006 (− 0.014 to 0.027)	0.521
Education (4–7 vs. lower)	1.095 (0.175 to 2.015)	0.020*	0.823 (− 0.134 to 1.781)	0.091	0.703 (− 0.745 to 2.152)	0.331
Tumor volume pre-op	− 0.001 (− 0.005 to 0.002)	0.453	0.003 (− 0.001 to 0.007)	0.189	0.010 (0.003 to 0.018)	0.009*
IDH1 (mutant vs. WT)	0.367 (− 0.083 to 0.817)	0.109			0.909 (0.172 to 1.647)	0.017*
1p19q deletie (+ vs. −)	− 0.281 (− 0.685 to 0.123)	0.169			− 0.530 (− 1.006 to − 0.053)	0.030*
WHO 2016 (+ vs. −)						
Gr. II/III IDH-WT. 1p19q (–)	− 0.612 (− 1.614 to 0.391)	0.230	− 0.871 (− 1.845 to 0.103)	0.079		
Gr. IV IDH-M	1.036 (− 0.134 to 2.205)	0.082	0.516 (− 0.766 to 1.797)	0.427		
Gr. IV IDH-WT	− 0.355 (− 0.855 to 0.145)	0.162	− 0.225 (− 0.909 to 0.459)	0.517		
Tumor location (+ vs. −)						
Left parietal	− 0.310 (− 0.810 to 0.189)	0.221	− 0.415 (− 0.994 to 0.163)	0.158	0.425 (− 0.282 to 1.133)	0.231
Left thalamus	− 1.336 (− 2.144 to − 0.528)	0.001*	− 1.329 (− 2.294 to − 0.365)	0.007*	− 0.087 (− 1.527 to 1.354)	0.904
Right frontal	− 0.314 (− 0.847 to 0.220)	0.247	− 0.566 (− 1.162 to 0.031)	0.063	− 0.374 (− 0.833 to 0.086)	0.108
Right temporal	− 0.463 (− 1.232 to 0.306)	0.236	− 0.403 (− 1.226 to 0.419)	0.334	− 0.435 (− 1.031 to 0.160)	0.146

*NCF* neurocognitive functioning, *ASA* American Society of Anaesthesiologists, *IDH1* isocitrate dehydrogenase 1, *WT* wildtype, *M* mutant, *1p19q* 1p19q deletion, *WHO* World Health Organization

*Significant (p < 0.05)

**Table 2:** Multivariable linear regression analyses for predicting delta-Z-scores (executive functioning)

Baseline variable	Univariable		Multivariable model	
			*R*^*2*^= 0.150 *p *= 0.009	
	B (95% CI)	p value	B (95% CI)	p value
Education (4–7 vs. lower)	0.454 (− 0.499 to 1.407)	0.248	0.159 (− 0.856 to 1.175)	0.757
Tumor volume pre-op	< 0.001 (− 0.004 to 0.003)	0.839	0.004 (− 0.001 to 0.009)	0.097
WHO 2016 (+ vs. −)				
Gr. II/III IDH-WT. 1p19q (–)	− 0.966 (− 1.979 to 0.047)	0.061	− 1.000 (− 2.031 to − 0.031)	0.057
Tumor location (+ vs. −)				
Both hemispheres	− 1.994 (− 3.486 to − 0.502)	0.009*	− 2.160 (− 3.673 to 0.648)	0.005 *
Left temporal	− 0.369 (− 0.833 to 0.096)	0.119	− 0.036 (− 0.529 to 0.456)	0.884
Left thalamus	− 1.011 (− 1.860 to − 0.162)	0.020*	− 1.319 (− 2.297 to − 0.342)	0.009*
Brainstem	− 1.321 (− 3.185 to 0.543)	0.163	− 0.500 (− 2.439 to 1.439)	0.61
First MRI (+ vs. −)				
Midlineshift	− 0.385 (− 1.007 to 0.238)	0.224	− 0.384 (− 1.185 to 0.417)	0.345

*NCF* neurocognitive functioning, *ASA* American Society of Anaesthesiologists, *IDH1* isocitrate dehydrogenase 1, *WT* wildtype, *M* mutant, *1p19q* 1p19q deletion, *WHO* World Health Organization

*Significant (p < 0.05)

**Table 3:** Multivariable linear regression analyses for predicting delta-Z-scores (psychomotor speed)

Baseline variable	Univariable		Multivariable model	
			*R*^*2*^= 0.200 *p *= 0.005	
	B (95% CI)	p value	B (95% CI)	p value
Age (at time of surgery)	− 0.011 (− 0.020 to − 0.002)	0.013*	− 0.005 (− 0.015 to 0.006)	0.394
Education				
Verhage 4–7 vs. lower	0.344 (− 0.227 to 0.915)	0.235	0.172 (− 0.407 to 0.750)	0.558
Tumor volume prior to surgery	0.000 (− 0.002 to 0.002)	0.819	0.001 (− 0.002 to 0.003)	0.517
WHO 2016 (+ vs. −)				
Gr. II/III IDH-M. 1p19q (–)	0.243 (− 0.080 to 0.565)	0.139	0.049 (− 0.292 to 0.390)	0.778
Gr. IV IDH-WT	− 0.293 (− 0.588 to 0.002)	0.052*	− 0.023 (− 0.447 to 0.401)	0.914
Tumor location (+ vs. −)				
Left insula	0.203 (− 0.060 to 0.466)	0.13	0.247 (− 0.030 to 0.523)	0.08
Left thalamus	− 0.702 (− 1.169 to − 0.236)	0.003*	− 0.827 (− 1.367 to − 0.288)	0.003*
First MRI (+ vs. −)				
Cystic	0.418 (0.053 to 0.782)	0.025*	0.447 (0.058 to 0.837)	0.025*
Enhancement	− 0.294 (− 0.551 to − 0.037)	0.025*	− 0.161 (− 0.505 to 0.183)	0.356
Necrosis	− 0.290 (− 0.574 to − 0.006)	0.045*	0.026 (− 0.374 to 0.427)	0.896

*NCF* neurocognitive functioning, *ASA* American Society of Anaesthesiologists, *IDH1* isocitrate dehydrogenase 1, *WT* wildtype, *M* mutant, *1p19q* 1p19q deletion, *WHO* World Health Organization

*Significant (p < 0.05)

**Table 4:** Multivariable linear regression analyses for predicting delta-Z-scores (visuospatial functioning)

Baseline variable	Univariable		Multivariable model	
			*R*^*2*^=0.372 *p *= 0.004	
	B (95% CI)	p value	B (95% CI)	p value
Male gender (+ vs. −)	0.325 (0.004 to 0.646)	0.048*	0.429 (0.066 to 0.793)	0.021*
ASA-score (1 vs. > 1)	0.342 (− 0.011 to 0.696)	0.058	0.304 (− 0.039 to 0.647)	0.082
Tumorvolume prior to surgery	0.001 (− 0.001 to 0.004)	0.278	− 0.004 (− 0.008 to − 0.001)	0.018*
WHO 2016 (+ vs. −)				
Gr. II/III IDH-M. 1p19q (–)	− 0.220 (− 0.593 to 0.153)	0.246	− 0.104 (− 0.565 to 0.356)	0.653
Gr. II/III IDH-WT. 1p19q (–)	− 0.594 (− 1.232 to 0.045)	0.068	− 0.374 (− 1.028 to 0.279)	0.258
Gr. IV IDH-M	0.511 (− 0.242 to 1.265)	0.182	0.230 (− 0.688 to 1.148)	0.619
Gr. IV IDH-WT	0.388 (0.039 to 0.737)	0.030*	0.285 (− 0.296 to 0.866)	0.331
Tumor location (+ vs. −)				
Left hemisphere	0.433 (0.091 to 0.774)	0.013*	0.432 (− 0.086 to 0.950)	0.101
Left insula	0.236 (− 0.068 to 0.540)	0.127	− 0.140 (− 0.580 to 0.300)	0.527
Left frontal	0.387 (0.091 to 0.683)	0.011*	0.200 (− 0.233 to 0.633)	0.36
Left parietal	0.286 (− 0.054 to 0.625)	0.099	− 0.109 (− 0.622 to 0.404)	0.673
Cortex involved	0.859 (0.041 to 1.678)	0.040*	0.468 (− 0.476 to 1.413)	0.327
Sulcus centralis	0.203 (− 0.099 to 0.505)	0.186	0.113 (− 0.383 to 1.608)	0.652
First MRI (+ vs. −)				
Cystic	0.322 (− 0.132 to 0.776)	0.162	0.096 (− 0.421 to 0.613)	0.713
Enhancement	0.294 (− 0.008 to 0.597)	0.056	− 0.087 (− 0.526 to 0.351)	0.693
Necrosis	0.403 (0.077 to 0.728)	0.016*	− 0.048 (− 0.589 to 0.494)	0.862
Midlineshift	0.769 (0.369 to 1.168)	< 0.001*	1.254 (0.619 to 1.889)	< 0.001*

*NCF* neurocognitive functioning, *ASA* American Society of Anaesthesiologists, *IDH1* isocitrate dehydrogenase 1, *WT* wildtype, *M* mutant, *1p19q* 1p19q deletion, *WHO* World Health Organization

*Significant (p < 0.05)

**Table 5:** Multivariable linear regression analyses for predicting delta-Z-scores (memory)

Baseline variable	Univariable		Multivariable model	
			*R*^*2*^=0.186 *p *=0.232	
	B (95% CI)	p value	B (95% CI)	p value
ASA-score (1 vs. > 1)	0.185 (− 0.032 to 0.403)	0.093	0.152 (− 0.079 to 0.383)	0.194
Tumorvolume prior to surgery	0.001 (0.000 to 0.003)	0.053	− 0.002 (− 0.004 to 0.001)	0.199
WHO 2016 (+ vs. −)				
Gr. II/III IDH-M. 1p19q (+)	− 0.184 (− 0.406 to 0.039)	0.105	0.034 (− 0.234 to 0.303)	0.799
Gr. IV IDH-M	0.536 (0.051 to 1.021)	0.031*	0.089 (− 0.516 to 0.695)	0.770
Tumor location (+ vs. −)				
Right hemisphere	− 0.211 (− 0.437 to 0.015)	0.067*	− 0.130 (− 0.534 to 0.275)	0.525
Left frontal	0.198 (0.006 to 0.391)	0.044*	0.080 (− 0.216 to 0.377)	0.591
Left parietal	0.166 (− 0.053 to 0.385)	0.136	0.033 (− 0.317 to 0.382)	0.853
Left insula	0.246 (0.052 to 0.439)	0.013*	0.042 (− 0.279 to 0.363)	0.797
Left hippocampus	0.317 (0.063 to 0.570)	0.015*	0.136 (− 0.245 to 0.517)	0.480
Multifocal	− 0.764 (− 1.846 to 0.317)	0.164	− 0.639 (− 1.749 to 0.471)	0.256
Sulcus centralis	0.139 (− 0.055 to 0.333)	0.159	0.168 (− 0.144 to 0.480)	0.286
First MRI (+ vs. −)				
Cystic	0.212 (− 0.068 to 0.492)	0.136	0.144 (− 0.191 to 0.480)	0.395
Oedema	0.236 (0.039 to 0.434)	0.019*	0.062 (− 0.256 to 0.381)	0.698
Necrosis	0.201 (− 0.007 to 0.408)	0.058	− 0.041 (− 0.358 to 0.276)	0.796
Midlineshift	0.377 (0.122 to 0.633)	0.004*	0.415 (− 0.018 to 0.848)	0.060

*NCF* neurocognitive functioning, *ASA* American Society of Anaesthesiologists, *IDH1* isocitrate dehydrogenase 1, *WT* wildtype, *M* mutant, *1p19q* 1p19q deletion, *WHO* World Health Organization

*Significant (p < 0.05)

**Table 6:** Multivariable linear regression analyses for predicting delta-Z-scores (language)

Baseline variable	Univariable		Multivariable model	
			*R*^*2*^=0.265 *p *≤ 0.001	
	B (95% CI)	p value	B (95% CI)	p value
Male gender (+ vs. −)	− 0.228 (− 0.613 to 0.158)	0.244	− 0.300 (− 0.651 to 0.051)	0.094
Tumor volume prior to surgery	0.003 (0.000 to 0.006)	0.024*	0.002 (− 0.002 to 0.005)	0.287
Tumor location (+ vs. −)				
Left temporal	− 0.405 (− 0.777 to − 0.033)	0.033*	− 0.206 (− 0.609 to 0.197)	0.314
Left thalamus	− 0.444 (− 1.155 to 0.267)	0.219	− 0.725 (− 1.441 to − 0.008)	0.048
Right frontal	0.350 (− 0.068 to 0.769)	0.1	0.150 (− 0.263 to 0.564)	0.473
Multifocal	− 3.528 (− 5.378 to − 1.678)	< 0.001*	− 3.220 (− 4.997 to − 1.443)	< 0.001*
Cortex involved	0.887 (− 0.244 to 2.017)	0.123	0.737 (− 0.306 to 1.779)	0.164
Sulcus centralis	0.295 (− 0.064 to 0.653)	0.106	0.085 (− 0.269 to 0.438)	0.637
First MRI (+ vs. −)				
Oedema	0.518 (0.155 to 0.882)	0.006*	0.411 (− 0.024 to 0.846)	0.064

*NCF* neurocognitive functioning, *ASA* American Society of Anaesthesiologists, *IDH1* isocitrate dehydrogenase 1, *WT* wildtype, *M* mutant, *1p19q* 1p19q deletion, *WHO* World Health Organization

*Significant (p < 0.05)

We used logistic regression analysis to evaluate baseline characteristics in relation to clinically relevant decline in NCF as a dichotomous outcome measure (deterioration of − 1 SD or lower versus > − 1 SD). The results of the univariable analyses are shown in Online Resource 4. Multivariable logistic regression analyses are shown in Tables 7, 8, 9, 10, 11, and 12.

**Table 7:** Multivariable logistic regression analyses (delta-Z-score = <− 1 SD vs. > − 1 SD) (overall neurocognitive functioning)

Baseline variable	Univariable		Multivariable	
	OR (95% CI)	p value	OR (95% CI)	p value
Age (at time of surgery)	1.110 (1.050 to 1.174)	< 0.001*	1.251 (1.035 to 1.511)	0.020*
Education (Verhage > 4)	0.192 (0.041 to 0.896)	0.036*	0.018 (0.000 to 0.819)	0.039*
ASA-score(> 2)	22.444 (1.851 to 272.176)	0.015*	20.888 (0.618 to 706.271)	0.091
Histology (glioblastoma)	8.455 (2.280 to 31.356)	0.001*	0.063 (0.001 to 4.172)	0.197
IDH1 (mutant)	0.079 (0.017 to 0.372)	0.001*	0.037 (0.001 to 1.611)	0.087
Tumorvolume preoperative T2	1.005 (0.998 to 1.012)	0.180	0.982 (0.962 to 1.003)	0.096
Tumorlocation				
Left thalamus	17.250 (4.167 to 71.410)	< 0.001*	83.824 (2.509 to 2800.980)	0.013*
Brainstem	7.867 (0.467 to 132.427)	0.152	0.318 (0.002 to 61.850)	0.67
First MRI				
Enhancement	3.989 (1.082 to 14.713)	0.038*	0.068 (0.002 to 2.175)	0.128
Necrosis	6.673 (2.145 to 20.758)	0.001*	3.701 (0.221 to 61.914)	0.363

*NCF* neurocognitive functioning, *ASA* American Society of Anaesthesiologists, *IDH1* isocitrate dehydrogenase 1, *WT* wildtype, *M* mutant, *1p19q* 1p19q deletion, *WHO* World Health Organization

*Significant (p < 0.05)

**Table 8:** Multivariable logistic regression analyses (delta-Z-score = < − 1 SD vs. > − 1 SD) (executive functioning)

Baseline variable	Univariable		Multivariable	
	OR (95% CI)	p value	OR (95% CI)	p value
Age (at time of surgery)	1.042 (1.000 to 1.086)	0.051	0.936 (0.836 to 1.047)	0.249
Education (Verhage > 4)	1.001 (0.999 to 1.004)	0.248	0.081 (0.001 to 5.098)	0.234
ASA-score (> 2)	4.500 (0.377 to 53.734)	0.235	3.312 (0.151 to 72.834)	0.448
Histology (glioblastoma)	2.567 (0.857 to 7.684)	0.092	1.557 (0.030 to 80.351)	0.826
IDH1 (mutant)	0.151 (0.040 to 0.577)	0.006*	0.013 (0.000 to 0.339)	0.009*
Tumorvolume preoperative T2	1.002 (0.994 to 1.010)	0.661	0.969 (0.939 to 1.000)	0.048*
Tumor location				
Both hemispheres	18.615 (1.578 to 219.569)	0.020*	161.265 (1.651 to 15748.513)	0.030*
Left frontal	6.720 (1.454 to 31.070)	0.015*	8.351 (0.873 to 79.924)	0.066
Left thalamus	4.036 (0.921 to 17.687)	0.064	30.700 (1.118 to 842.879)	0.043*
Brainstem	8.500 (0.503 to 143.546)	0.138	1.241 (0.007 to 211.880)	0.934
First MRI				
Enhancement	2.412 (0.728 to 7.994)	0.150	0.255 (0.013 to 4.978)	0.368
Necrosis	3.082 (1.036 to 9.169)	0.043*	0.229 (0.013 to 4.103)	0.316
Midline shift	2.101 (0.603 to 7.326)	0.244	25.160 (0.179 to 3530.628)	0.201

*NCF* neurocognitive functioning, *ASA* American Society of Anaesthesiologists, *IDH1* isocitrate dehydrogenase 1, *WT*  wildtype, *M* mutant, *1p19q* 1p19q deletion, *WHO* World Health Organization

*Significant (p < 0.05)

**Table 9:** Multivariable logistic regression analyses (delta-Z-score = < − 1 SD vs. > − 1 SD) (psychomotor speed)

Baseline variable	Univariable		Multivariable	
	OR (95% CI)	p value	OR (95% CI)	p value
Age (at time of surgery)	1.047 (1.008 to 1.088)	0.017*	0.994 (0.932 to 1.061)	0.855
Gender (male)	0.336 (0.092 to 1.226)	0.098	0.214 (0.032 to 1.427)	0.111
Education (Verhage > 4)	0.434 (0.078 to 2.419)	0.341	0.391 (0.028 to 5.529)	0.487
ASA-score (> 1)	2.135 (0.638 to 7.141)	0.218	1.221 (0.273 to 5.458)	0.794
WHO2016				
Gr. II/III IDH-M. 1p19q (–)	0.164 (0.021 to 1.289)	0.086	0.325 (0.024 to 4.385)	0.397
Gr. IV IDH-WT	4.511 (1.597 to 12.738)	0.004*	1.699 (0.241 to 11.987)	0.595
Tumor volume preoperative T2	1.001 (0.994 to 1.009)	0.761	0.989 (0.975 to 1.003)	0.116
Tumor location				
Left hemisphere	2.105 (0.572 to 7.752)	0.263	0.354 (0.023 to 5.428)	0.456
Left thalamus	7.143 (1.833 to 27.828)	0.005*	20.564 (1.625 to 260.212)	0.020*
Right frontal	0.340 (0.074 to 1.568)	0.167	0.459 (0.025 to 8.291)	0.598
First MRI				
Enhancement	5.752 (1.582 to 20.908)	0.008*	2.189 (0.334 to 14.331)	0.414
Necrosis	3.419 (1.253 to 9.325)	0.016*	0.915 (0.157 to 5.345)	0.922

*NCF* neurocognitive functioning, *ASA* American Society of Anaesthesiologists, *IDH1*  isocitrate dehydrogenase 1, *WT*  wildtype, *M* mutant, *1p19q*  1p19q deletion, *WHO* World Health Organization

*Significant (p < 0.05)

**Table 10:** Multivariable logistic regression analyses (delta-Z-score = < − 1 SD vs. > − 1 SD) (visuospatial functioning)

Baseline variable	Univariable		Multivariable	
	OR (95% CI)	p value	OR (95% CI)	p value
ASA-score (> 1)	0.498 (0.173 to 1.435)	0.197	0.337 (0.095 to 1.190)	0.091
WHO2016				
Gr. II/III IDH-WT. 1p19q (–)	6.741 (1.388 to 32.727)	0.018*	10.194 (1.475 to 70.474)	0.019*
Tumorvolume preoperative T2	0.997 (0.989 to 1.005)	0.455	1.007 (0.994 to 1.020)	0.278
Tumor location				
Right hemisphere	2.286 (0.838 to 6.236)	0.106	0.799 (0.118 to 5.421)	0.818
Left frontal	0.266 (0.095 to 0.740)	0.011*	0.152 (0.027 to 0.844)	0.031*
Left insula	0.553 (0.207 to 1.482)	0.239	1.605 (0.308 to 8.374)	0.574
Right parietal	2.389 (0.649 to 8.795)	0.19	1.576 (0.204 to 12.206)	0.663
First MRI				
Necrosis	0.459 (0.143 to 1.471)	0.19	0.357 (0.064 to 1.978)	0.238

*NCF* neurocognitive functioning, *ASA* American Society of Anaesthesiologists, *IDH1* isocitrate dehydrogenase 1, *WT* wildtype, *M* mutant, *1p19q* 1p19q deletion, *WHO* World Health Organization

*Significant (p < 0.05)

**Table 11:** Multivariable logistic regression analysis; memory

Variables were selected for multivariable logistic regression analysis if a regression equation was found with a p value of < 0.25 in univariable analysis. No variables were found with a p value < 0.25 in univariable logistic regression analyses

**Table 12:** Multivariable logistic regression analyses (delta-Z-score = <− 1 SD vs. > − 1 SD) (language)

Baseline variable	Univariable		Multivariable	
	OR (95% CI)	p value	OR (95% CI)	p value
Age (at time of surgery)	1.039 (0.990 to 1.091)	0.121	0.938 (0.838 to 1.050)	0.268
Education (Verhage > 4)	0.158 (0.025 to 1.001)	0.050*	0.553 (0.015 to 21.024)	0.749
Histology (glioblastoma)	7.270 (2.107 to 141.577)	0.008*	78.084 (0.977 to 6240.664)	0.051
IDH1 (mutant)	0.071 (0.009 to 0.594)	0.015*	0.857 (0.010 to 71.155)	0.946
Tumorvolume preoperative T2	1.004 (0.994 to 1.014)	0.414	0.987 (0.962 to 1.013)	0.314
Tumor location				
Left temporal	7.618 (1.503 to 38.612)	0.014*	8.263 (0.581 to 117.556)	0.119
Left hippocampus	3.100 (0.699 to 13.746)	0.137	0.644 (0.044 to 9.480)	0.749
Left thalamus	4.857 (0.824 to 28.633)	0.081	5.650 (0.249 to 128.082)	0.277
Right occipital	6.625 (0.541 to 81.172)	0.139	20.134 (0.273 to 1485.592)	0.171
Cortex involved	0.151 (0.012 to 1.849)	0.139	0.063 (0.001 to 3.397)	0.174
First MRI				
Necrosis	6.356 (1.540 to 26.241)	0.011*	0.218 (0.011 to 4.420)	0.321
Midline shift	3.833 (0.972 to 15.116)	0.055	5.878 (0.078 to 443.831)	0.422

*NCF* neurocognitive functioning, *ASA* American Society of Anaesthesiologists, *IDH1* isocitrate dehydrogenase 1, *WT* wildtype, *M* mutant, *1p19q* 1p19q deletion, *WHO* World Health Organization

*Significant (p < 0.05)

Figure 3 depicts the significant (p < 0.05) results of both the linear regression analyses for cognitive changes, and the logistic regression analysis for cognitive deterioration.

## Discussion

Through this retrospective study we give an overview of changes in NCF in patients with a diffuse glioma after surgery and initial other anti-tumor treatment and show which factors are of influence on cognitive changes after surgery.

Our results show that the domain psychomotor speed was most vulnerable to the effects of surgery, both on the group level and for the proportion of individual-level deficits. Visuospatial functioning significantly deteriorated after surgery on the group level. In the subgroup of patients who did not receive adjuvant treatment after surgery, results were numerically similar to the whole study sample, underlining that our whole-group results are unlikely to be biased by the effects of radiotherapy or chemotherapy. Surgery (and other therapies) seem to be of greater influence on cognitive functioning in LGG than in HGG patients.

These results support our hypothesis that neurocognitive functioning is mostly maintained after awake surgery across different domains.

This is somewhat in contrast with the findings of Incekara et al., who observed a deterioration in language functions after awake surgery of eloquently located presumed LGGs. Their smaller-scale study was mostly focused at left hemisphere brain regions, whereas our results represent a wider range of glioma patients [14]. Satoer et al., also found postoperative decline in language and executive functions. These differences with our study results can probably be explained by the benefits of awake surgery in our study [7]. In more detail, neurocognitive functioning appears harder to preserve in the domain psychomotor speed. This domain is dependent on widespread cerebral networks rather than on specific brain regions [15]. In a previous study we found that “location-independent” domains are affected most in treatment-naive glioma patients [2]. Consistent with this previous finding on baseline functioning, we now observe that psychomotor speed also is more vulnerable than other domains to the effects of surgery, other types of therapy (radiotherapy or chemotherapy) and the initial course of the disease.

In subgroup analysis, more LGG patients had cognitive decline after surgery than in HGG patients. Again, this was especially found in the domain psychomotor speed. This finding underscores the vulnerability of LGG patients, who have less cognitive deficits prior to surgery than HGG patients, so probably have more to lose regarding cognition. Another possible explanation for this finding is that HGG patients have more beneficial effect of mass reduction during surgery due to more edema, cystic/necrotic mass lesions and greater overall tumor volume; indeed, pre-operative midline shift, tumor volume and cystic tumor components turned out to predict postoperative cognitive recovery in our analysis.

Extension of the tumor into the left thalamus was a significant predictor of cognitive decline for overall-NCF, executive functioning and psychomotor speed. About ten percent of the study sample had such thalamic involvement on pre-operative T2/FLAIR MRI. Most of these “thalamic lesions” were HGG (11/16). In HGG patients, thalamic involvement most commonly consisted of edema, whereas thalamus involvement in 3 of 5 LGGs was caused by the tumor itself. In none of these patients, the thalamus was resected.

The thalamus, with its cortical, subcortical and cerebellar connections, is known to be a critical node in functional brain networks supporting cognitive functions [16, 17]. Thereby, thalamic lesions can have global and distal effects on cortical network organization [17].

Speculatively, edema and/or infiltrative growth in this region pre-operatively already causes irreversible damage and thereby lessens chance of good cognitive recovery, especially in domains which rely on a widespread network. Another explanation for the negative influence of thalamic involvement on cognitive recovery can be that “thalamic gliomas” form a distinct subgroup of tumors with a specific molecular profile. Such regional variation in glioma biology was illustrated recently by Zhou et al. [18].

Other structural effects we found were a negative influence of larger pre-operative tumor volume and a positive influence of midline-shift on the domain visuospatial functioning. These combined findings suggest that neuropsychological deficits that develop as a result of increased intracranial pressure of the whole brain (represented by midline-shift), are different and more reversible than deficits that arise by direct invasion of the tumor (as represented by pre-operative volume). This hypothesis also explains why cystic lesions were associated with postoperative improvement in the domain psychomotor speed: by operating a cystic tumor, intracranial pressure decreases, resulting in a positive effect on this “location-independent” domain.

In the domain executive functioning, larger pre-operative tumor volume was associated with cognitive improvement, which may—again—reflect the positive effect of mass reduction during surgery. In addition to such a structural effect, we found that the classification of the tumor as a grade II/III 1p19q-intact and IDH-wildtype glioma predicted cognitive decline in visuospatial functioning. Also, an IDH-mutation predicted favorable recovery in executive functioning. A possible explanation for this finding is that the molecular profile of tumors results in biochemical changes in the surrounding (and possibly distant) brain parenchyma, with changes in brain functioning, postoperative plasticity and—ultimately—neurocognitive functioning as a consequence. This hypothesis is supported by a recent study by Wefel et al., who found a complex interrelationship between patients’ NCF, tumor growth velocity and the presence or absence of an IDH-mutation [19, 20].

Besides the effect of molecular markers on certain genetic pathways, the slower growth of IDH-mutated tumors can be a possible explanation for better cognitive recovery. Further research into additional associations between tumor biology on the one hand, and neurocognitive functioning and recovery on the other, is needed so anti-tumor treatment can be maximized with minimal adverse effects on NCF.

A limitation of our study is that most (> 70%) patients received additional therapy after surgery before post-operative neuropsychological assessment was performed. This timing of post-operative neuropsychological assessment was chosen because too early evaluation may be confounded by incomplete postoperative recovery (e.g. edema, supplementary motor area (SMA) syndrome). Since radiotherapy and chemotherapy [21] may influence cognitive functioning, effects of adjuvant therapy cannot be distinguished from the sequelae of surgery. However, our results form a valid representation of the postoperative course of NCF in glioma patients receiving awake brain tumor surgery in or near eloquent brain regions, and standard-of-care adjuvant treatment. Also, subgroup analysis for patients who did not receive any therapy after surgery mostly confirmed the findings from whole-group analyses, suggesting that the influence of chemo- and radiotherapy was limited. In line with this, we also did not correct for extent of resection. Altogether, we feel that our data reflect the neurocognitive effects of standard-of-care glioma surgery aimed at maximum extent of resection with preservation of brain function.

Secondly, we only included patients in our study sample who underwent awake surgery. Our results are thus primarily generalizable to this specific group of patients, with relatively good clinical performance and a predilection for certain localizations of the tumor [22]. In addition, the percentage of LGG patients is higher in the group of awake surgery patients than in the total glioma population, underscoring that our findings are primarily applicable to in NCF changes after awake surgery.

Selective loss of patients for post-operative assessment (< 20% of all eligible patients) played a role. Most patients who did not undergo post-operative testing were in clinically bad condition, had already died or refused neuropsychological assessments. Probably these results lead to an underestimation of the real number of patients with postoperative decline in NCF.

Of note, we decided to group tasks on their conceptual background (‘domain’) in order to enhance power. Such grouping is always complicated since intrinsically more than one concept is tapped in any task. However, neuropsychologists do share common ground in the categorization of tasks across domains [23–25]. We tested for robustness of our test classification by applying an alternative grouping of tests, which only resulted in minimal changes in outcomes.

Analysis of difference scores (before versus after) carries the risk of regression to the mean, especially at the extremes of the spectrum of cognitive scores; we checked for this by repeating group-level analyses for executive functioning and memory, with omission of the most extreme pre-operative Z-values. These sensitivity analyses did not show significant results (mean delta executive functioning 0.0915; independent sample *t* test p = 0.225 and mean delta memory 0.019; independent sample t test p = 0.755).

Strengths of this study include the large sample size and the extensiveness of data on pre- and postoperative NCF. We systematically reported on therapies that could have been of influence on cognitive changes and performed subgroup analysis in a study sample who did not receive any treatment after surgery. Of note, all neuropsychological data were prospectively collected and tested according to a standard clinical procedure leading to a homogeneous set of neuropsychological tasks.

## Conclusion

In patients undergoing awake surgery for a diffuse glioma, cognitive functioning declines after surgery in the domains of visuospatial functioning and psychomotor speed, but not in other domains. It can, therefore, be valuable to pay specific attention to these domains during awake surgery in addition to the more commonly evaluated domains such as language.

Involvement of the thalamus, larger tumor-volume and IDH-mutation were the most important determinants of cognitive outcome after awake surgery. These results underline that a combination of structural and biomolecular effects from the tumor determine postoperative cognitive performance. Deeper knowledge of tumor-genetic markers that predict neurocognitive changes after surgery is necessary and will likely facilitate the development of new strategies for patient counseling as well as treatment and rehabilitation.

## Electronic supplementary material

Below is the link to the electronic supplementary material.

Electronic supplementary material 1: Online Resource 1 (DOC 89 kb) STROBE criteria

Electronic supplementary material 2: Online Resource 2 (DOCX 31 kb) Supplementary methods

Electronic supplementary material 3: Online Resource 3 (DOCX 45 kb) Determinants of cognitive change after surgery: univariable linear regression analyses

Electronic supplementary material 4: Online Resource 4 (DOCX 45 kb) Determinants of cognitive decline (decrease of Z-score of 1 or more) after surgery: univariable logistic regression analyses

Electronic supplementary material 5: Online Resource Table 1 (DOCX 21 kb) Multivariable linear regression analyses for predicting delta-Z-scores

Electronic supplementary material 6: Online Resource Table 2 (DOCX 17 kb) Neuropsychological tasks per domain

Electronic supplementary material 7: Online Resource Fig. 1 (EPS 816 kb) Reasons for not undergoing postoperative neuropsychological assessment

Electronic supplementary material 8: Online Resource Fig. 2 (EPS 861 kb) Individual level analyses — distribution of alterations in NCF 3–6 months post-operatively

Electronic supplementary material 9: Online Resource Fig. 3 (EPS 2316 kb) Subgroup analyses of cognitive postoperative change on group level **a** Patients who did not undergo postoperative adjuvant treatment **b** Low-grade glioma **c** High-grade glioma. *Wilcoxon related sample test performed, because these data was not normally distributed

Electronic supplementary material 10: Online Resource Fig. 4 (EPS 1136 kb) Group-level analyses of pre- and postoperative scores (Z-scores) per domain – boxplots with median, 25th and 75th percentiles. O = outlier

Electronic supplementary material 11: Online Resource Fig. 5 (EPS 1427 kb) Subgroup analyses of cognitive postoperative change on individual level **a** patients who did not undergo postoperative adjuvant treatment **b** low-grade glioma; **c** high-grade glioma
