# Supplementary material for: Neurocognitive changes after awake surgery in glioma patients: a retrospective cohort study
Source: J Neurooncol. 2019 Dec 4;146(1):97–109. doi: 10.1007/s11060-019-03341-6 (PMC6938472; doi:10.1007/s11060-019-03341-6)
Supplement: Supplementary file 2 — Electronic supplementary material 2: Online Resource 2 (DOCX 31 kb) Supplementary methods [file 11060_2019_3341_MOESM2_ESM.docx]

**Title: Neurocognitive changes after awake surgery in glioma patients: A retrospective cohort study
Journal:** Journal of Neuro-Oncology

**Authors:** Emma van Kessel MD*, Tom J. Snijders MD PhD, Anniek E. Baumfalk MD, Carla Ruis PhD, Kirsten M. van Baarsen MD PhD, Marike L. Broekman MD PhD, Martine J.E. van Zandvoort PhD**, Prof. Pierre A. Robe MD PhD**

**Affiliations and email of corresponding author:**
University Medical Center Utrecht/UMC Utrecht Brain Center, Department of Neurology & Neurosurgery, internal adress G03.232, PO Box 85500, 3508 XC Utrecht, The Netherlands. e-mail: [e.vankessel-2@umcutrecht.nl](mailto:e.vankessel-2@umcutrecht.nl)

**Online Resource 2:** Supplementary methods

*Participants*

Data were obtained from a consecutive cohort of patients with diffuse gliomas at the University Medical Center Utrecht, The Netherlands (UMCU) who underwent awake neurosurgery. Almost all patients who underwent awake brain tumor surgery were seen for pre- and post-operative elaborate neuropsychological testing. Inclusion criteria for this study were the presence of a diffuse glioma (WHO grades 2-4) according to the criteria of the World Health Organization (WHO, 2016) and a minimum age of eighteen years. All patients were operated under awake conditions, as described below. Exclusion criteria were (a) any form of tumor-directed treatment (operation, chemotherapy, radiotherapy) before *pre-operative* neuropsychological assessment and (b) insufficient neuropsychological assessment, as was rarely the case due to emergency surgery or for pure motor deficits in centrally located tumors. Data was considered insufficient for a certain domain if less than 50% of tasks within that domain were performed, and insufficient overall if data on less than four domains were obtained. Patients who underwent initial antineoplastic treatment, including standard-of-care chemo- and/or radiotherapy, *between* surgery and the postoperative neuropsychological assessment were still eligible for inclusion; by including these patients, we aimed to reflect the changes in NCF after glioma surgery in a realistic context. Also, postoperative testing of NCF after glioma surgery is ideally performed after several months wherein initial cognitive recovery may occur [1], and clinically indicated adjuvant treatment could not be withheld from the patients for ethical reasons. Data about the details of post-operative treatment were carefully collected so that subgroup analysis could be performed.

*Surgical procedure and post-operative treatment*

Selection of patients for awake procedure was done as part of routine clinical care, and was based on whether the tumor was located in or near an eloquent brain area.

Decisions about post-operative treatment were made in institutional multidisciplinary tumor board, in accordance with Dutch national guidelines. Accordingly, clinical criteria such as tumor grade, molecular markers, extent of resection, age and clinical condition of the patient were considered in the decision process. Post-operative treatment started within 6 weeks after operation.

*Neuropsychological tests and assessment*

Included patients received a pre- and post-operative neuropsychological test battery as a part of routine clinical care. The neuropsychological tests selected (see Online Resource table 2) are widely internationally used, standardized psychometric instruments for assessing neurocognitive deficits in all major neurocognitive domains. All have normative data that take into account age and, when appropriate, gender and educational level. At repeated (postoperative) testing, we made use of alternate forms whenever possible to minimize practice effects.

Neuropsychological tasks often tap more than one cognitive domain and classification into cognitive domains often differs in the literature. We made use of a predetermined test classification based on previous literature [2-6] (Online Resource table 2). To test for robustness of our neuropsychological test classification, we repeated our analyses while re-classifying the tasks “Digit Span Backward” and “Semantic Fluency” as part of executive functioning instead of memory.

Classifying the task “Digit Span Backward” as part of executive functioning did not result in changes in the percentage of impairments, when compared to our original analysis. A second variant, classifying both tasks “Digit Span Backward” and “Semantic Fluency” as part of executive functioning, results in minimal changes of the proportion of patients with impairments, with absolute changes for different domains in the range of 0.7-2.4%. On the group level, the two variants of test classification results in minimal changes in mean Z-scores (less than 0.03 for all domains).

The pre-operative neuropsychological evaluation was conducted shortly (1-7 days) before the awake brain tumor surgery and the post-operative neuropsychological evaluation 3-6 months after surgery, depending on tumor grade: 3 months for high-grade gliomas because of the associate short life expectancy and chance of early deterioration, and 6 months for low-grade. Most of the patients with a clinical indication for post-operative radiotherapy and/or chemotherapy had either just completed this treatment at the moment of post-operative neuropsychological assessment or were still receiving such treatment. All evaluations were performed with use of standardized test instructions and under supervision of a clinical neuropsychologist. The neuropsychological evaluation took most patients approximately two hours to complete. Patients were tested under optimal testing conditions with at least one break inserted halfway, to make the test battery less exhausting.

*Patients and tumor characteristics*

Determinants we collected included data on tumor grade and molecular markers, which were converted into the WHO 2016 classification [13]. IDH-status was determined with next-generation sequencing (NGS), or immunohistochemical evaluation for the IDH1 R132H mutation. 1p/19q codeletion, was measured with multiplex ligation-dependent probe amplification or NGS. To obtain data about glioma location, lateralization and volume, for each patient the pre-operative MRI (T2/fluid attenuation inversion recovery (FLAIR) and T1 after administration of gadolinium) was viewed and measured by a junior clinical scientist (EvK) and reviewed by an experienced neuro-oncologist (TJS). Volumes were measured in 3D with use of Osirix Lite (v. 9.5.2) on T2-/fluid-attenuated inversion recovery (FLAIR)-weighted MRI scans and the volume was defined as the whole area of hyperintensity. This represents the total lesion volume, including tumor infiltration and edema. Since this parameter is independent of enhancement (and thereby grade) of the lesion, it forms a widely usable representation of the extent of brain volume that is potentially hampered in its function by the tumor in any way.

Involvement of the glioma in frontal, parietal, temporal, occipital, hippocampus, insula, thalamus, cortex, central sulcus, brainstem and multifocal regions of the brain were registered.

*Analysis*

*Group-level:* An individual patient’s Z-score for a given cognitive domain was calculated as the mean of the patient’s Z-scores derived from all tasks in this domain. Based on these individual mean Z-scores, group domain scores were calculated for the following neurocognitive domains: executive functioning and attention, memory, language, visuospatial functioning and processing speed. An overall NCF was also computed as the mean of the Z-scores of all of the five neurocognitive domains.

*Individual-level:* To determine the percentage of impaired patients at the domain level, we counted the number of *individual* patients with impairment-level scores per domain, before and after surgery. A patient was considered impaired for the given domain if he or she performed below threshold (-2SD) on any of the administered tests within the domain [7].

For both analyses (group and individual), we performed complete-case analysis; we decided not to impute for missing data because this only concerned a small percentage of data (1-10% for the different domains).

*Determinants of influence on changes in NCF*

We evaluated the predictive value of baseline characteristic (before surgery) on change in NCF, using both univariable and multivariable linear regression analysis. Outcome measures were the 6 delta-Z-scores of the individual patient (overall NCF and 5 different domains). Variables were selected for multivariable linear regression analysis if a regression equation was found with a p-value of <0.25 in univariable analysis. To avoid multicollinearity, we calculated correlation coefficients between all determinants. We selected variables for multivariable analysis with correlation coefficients <0.4. In addition, we used logistic regression analysis to evaluate the association between baseline characteristics and the dichotomous outcome measure of clinically relevant decline in NCF (decrease of Z-score of 1 or more). Variables were selected for multivariable logistic regression analyses according to the same criteria as in the linear regression analyses (univariable p<0.25).

**References**

1. Satoer D, Visch-Brink E, Smits M, Kloet A, Looman C, Dirven C, Vincent A (2014) Long-term evaluation of cognition after glioma surgery in eloquent areas. J Neurooncol 116 (1):153-160. doi:10.1007/s11060-013-1275-3

2. van Kessel E, Baumfalk AE, van Zandvoort MJE, Robe PA, Snijders TJ (2017) Tumor-related neurocognitive dysfunction in patients with diffuse glioma: a systematic review of neurocognitive functioning prior to anti-tumor treatment. J Neurooncol 134 (1):9-18. doi:10.1007/s11060-017-2503-z

3. Doherty JM, Belletier C, Rhodes S, Jaroslawska A, Barrouillet P, Camos V, Cowan N, Naveh-Benjamin M, Logie RH (2018) Dual-task costs in working memory: An adversarial collaboration. J Exp Psychol Learn Mem Cogn. doi:10.1037/xlm0000668

4. Biesbroek JM, van Zandvoort MJ, Kappelle LJ, Velthuis BK, Biessels GJ, Postma A (2016) Shared and distinct anatomical correlates of semantic and phonemic fluency revealed by lesion-symptom mapping in patients with ischemic stroke. Brain Struct Funct 221 (4):2123-2134. doi:10.1007/s00429-015-1033-8

5. Nys GM, van Zandvoort MJ, de Kort PL, Jansen BP, de Haan EH, Kappelle LJ (2007) Cognitive disorders in acute stroke: prevalence and clinical determinants. Cerebrovasc Dis 23 (5-6):408-416. doi:10.1159/000101464

6. Berendsen S, Varkila M, Kroonen J, Seute T, Snijders TJ, Kauw F, Spliet WG, Willems M, Poulet C, Broekman ML, Bours V, Robe PA (2016) Prognostic relevance of epilepsy at presentation in glioblastoma patients. Neuro Oncol 18 (5):700-706. doi:10.1093/neuonc/nov238

7. Palmer BW, Boone KB, Lesser IM, Wohl MA (1998) Base rates of "impaired" neuropsychological test performance among healthy older adults. Arch Clin Neuropsychol 13 (6):503-511
