# Supplementary material for: Neurocognitive changes after awake surgery in glioma patients: a retrospective cohort study
Source: J Neurooncol. 2019 Dec 4;146(1):97–109. doi: 10.1007/s11060-019-03341-6 (PMC6938472; doi:10.1007/s11060-019-03341-6)
Supplement: Supplementary file 4 — Electronic supplementary material 4: Online Resource 4 (DOCX 45 kb) Determinants of cognitive decline (decrease of Z-score of 1 or more) after surgery: univariable logistic regression analyses [file 11060_2019_3341_MOESM4_ESM.docx]

**Title: Neurocognitive changes after awake surgery in glioma patients: A retrospective cohort study
Journal:** Journal of Neuro-Oncology

**Authors:** Emma van Kessel MD*, Tom J. Snijders MD PhD, Anniek E. Baumfalk MD, Carla Ruis PhD, Kirsten M. van Baarsen MD PhD, Marike L. Broekman MD PhD, Martine J.E. van Zandvoort PhD**, Prof. Pierre A. Robe MD PhD**

**Affiliations and email of corresponding author:**
University Medical Center Utrecht/UMC Utrecht Brain Center, Department of Neurology & Neurosurgery, internal adress G03.232, PO Box 85500, 3508 XC Utrecht, The Netherlands. e-mail: [e.vankessel-2@umcutrecht.nl](mailto:e.vankessel-2@umcutrecht.nl)

**Online Resource 4: Univariable logistic regression analyses (delta-Z-score <-1SD of >-1SD)**

1. Univariable logistic regression analyses: overall neurocognitive functioning

| Baseline variable | Univariable | |
| --- | --- | --- |
|  |  |  |
|  | **OR (95% CI)** | **p-value** |
|  |  |  |
| Age (at time of surgery) | 1.110 (1.050 – 1.174) | 0.000 |
| Gender (male) | 1.311 (0.444 – 3.869) | 0.624 |
| Education  Verhage 6-7 vs lower  Verhage 5-7 vs lower  Verhage 4-7 vs lower  Verhage 3-7 vs lower | 0.382 (0.116 – 1.253)  0.717 (0.230 – 2.235)  0.192 (0.041 – 0.896)  0.259 (0.022 – 3.027) | 0.112  0.566  0.036  0.281 |
| ASA-score  >2 vs lager  >1 vs lager | 22.444 (1.851 – 272.176)  3.150 (0.647 – 15.325) | 0.015  0.155 |
| Tumor grade (HGG vs LGG) | 0.997 (0.997 – 1.018) | 0.800 |
| Histology  Astrocytoma  Oligodendroglioma  Mixed glioma  Glioblastoma  Ganglioglioma | 0.000 (0.000 – 0.000)  1.547 (0.169 – 14.146)  0.300 (0.065 – 1.387)  8.455 (2.280 – 31.356)  0.000 (0.000- 0.000) | 0.999  0.699  0.123  0.001  0.999 |
| IDH1 (Mutant vs WT) | 0.079 (0.017 – 0.372) | 0.001 |
| 1p19q deletion (+ vs absent) | 0.944 (0.080 – 11.152) | 0.964 |
| WHO 2016 (+ vs -)  Gr. II/III IDH-M. 1p19q (-)  Gr. II/III IDH-M. 1p19q (+)  Gr. II/III IDH-WT. 1p19q (-)  Gr. IV IDH-M  Gr. IV IDH-WT | 0.000 (0.000 – 0.000)  0.424 (0.091 – 1.974)  1.256 (0.141 – 11.156)  0.000 (0.000 – 0.000) 10.731 (3.193 – 36.066) | 0.998  0.274  0.838  0.999  0.000 |
| Volume pre-operatief (T2 FLAIR) | 1.005 (0.998 – 1.012) | 0.180 |
| Tumor location (involvement + vs -)  Left hemisphere  Right hemisphere  Both hemispheres  Left frontal  Left parietal  Left temporal  Left occipital  Left insula  Left hippocampus  Left thalamus  Right frontal  Right parietal  Right temporal  Right occipital  Right insula  Right hippocampus  Right thalamus  Multifocal  Cortical involvement  Brainstem  Sulcus Centralis | 1.033 (0.310 – 3.441)  1.107 (0.331 – 3.705)  0.000 (0.000 – 0.000)  0.859 (0.302 – 2.441)  1.862 (0.623 – 5.567) 1.767 (0.619 – 5.045)  1.731 (0.438 – 6.836)  0.547 (0.179 – 1.672)  1.650 (0.483 – 5.641)  17.250 (4.167 – 71.410)  1.192 (0.355 – 4.007)  0.544 (0.066 – 4.459)  1.403 (0.281 – 6.990)  2.578 (0.252 – 26.393)  0.990 (0.205 – 4.796)  0.000 (0.000 – 0.000)  0.000 (0.000 – 0.000)  0.000 (0.000 – 0.000)  0.522 (0.055 – 4.982)  7.867 (0.467 – 132.427)  1.125 (0.396 – 3.196) | 0.957  0.869  0.999  0.776  0.266  0.287  0.434  0.290  0.452  0.000  0.776  0.570  0.680  0.452  0.991  0.999  0.999  1.000  0.572  0.152  0.825 |
| First MRI (+ vs -)  Cystic  Oedema  Enhancement  Necrosis  Midlineshift | 0.938 (0.195 – 4.516)  1.518 (0.527 – 4.367)  3.989 (1.082 – 14.713)  6.673 (2.145 – 20.758)  1.907 (0.553 – 6.574) | 0.936  0.439  0.038  0.001  0.306 |
|  |  |  |

1. Univariable logistic regression analyses; executive functioning

| Baseline variable | Univariable | |
| --- | --- | --- |
|  |  |  |
|  | **OR (95% CI)** | **p-value** |
|  |  |  |
| Age (at time of surgery) | 1.042 (1.000 – 1.086) | 0.051 |
| Gender (male) | 1.064 (0.341 – 3.324) | 0.915 |
| Education  Verhage 6-7 vs lower  Verhage 5-7 vs lower  Verhage 4-7 vs lower  Verhage 3-7 vs lower | 1.308 (0.432 – 3.961)  2.182 (0.463 – 10.286)  1.001 (0.999 – 1.004)  1.001 (0.999 – 1.004) | 0.635  0.324  0.248  0.247 |
| ASA-score  >2 vs lager  >1 vs lager | 4.500 (0.377 – 53.734)  2.050 (0.532 – 8.033) | 0.235  0.303 |
| Tumor grade (HGG vs LGG) | 0.996 (0.996 – 1.039) | 0.850 |
| Histology  Astrocytoma  Oligodendroglioma  Mixed glioma  Glioblastoma  Ganglioglioma | 1.603 (0.507 – 5.072)  1.671 ( 0.182 – 15.350)  0.000 (0.000 – 0.000)  2.567 (0.857 – 7.684)  0.000 (0.000 – 0.000) | 0.422  0.650  0.998  0.092  0.999 |
| IDH1 (Mutant vs WT) | 0.151 (0.040 – 0.577) | 0.006 |
| 1p19q deletion (+ vs absent) | 0.216 (0.018 – 2.559) | 0.224 |
| WHO 2016 (+ vs -)  Gr. II/III IDH-M. 1p19q (-)  Gr. II/III IDH-M. 1p19q (+)  Gr. II/III IDH-WT. 1p19q (-)  Gr. IV IDH-M  Gr. IV IDH-WT | 0.483 (0.103 – 2.266)  0.205 (0.026 – 1.624)  3.538 (0.623 – 20.104)  0.000 (0.000 – 0.000)  4.707 (1.544 – 14.345) | 0.356  0.133  0.154  0.999  0.006 |
| Volume pre-operatief (T2 FLAIR) | 1.002 (0.994 – 1.010) | 0.661 |
| Tumor location (involvement + vs -)  Left hemisphere  Right hemisphere  Both hemispheres  Left frontal  Left parietal  Left temporal  Left occipital  Left insula  Left hippocampus  Left thalamus  Right frontal  Right parietal  Right temporal  Right occipital  Right insula  Right hippocampus  Right thalamus  Multifocal  Cortical involvement  Brainstem  Sulcus Centralis | 0.937 (0.278 – 3.157)  0.472 (0.101 – 2.211)  18.615 (1.578 – 219.569)  6.720 (1.454 – 31.070)  2.092 (0.686 – 6.375)  1.111 (0.371 – 3.328)  1.077 (0.221 – 5.249)  1.546 (0.527 – 4.540)  0.685 (0.144 – 3.258)  4.036 (0.921 – 17.687)  0.861 (0.226 – 3.275)  0.588 (0.071 – 4.844)  0.643 (0.078 – 5.327)  0.000 (0.000 – 0.000)  1.077 (0.221 – 5.249)  0.000 (0.000 – 0.000)  0.000 (0.000 – 0.000)  0.000 (0.000 – 0.000)  0.000 (0.000 – 0.000)  8.500 (0.503 – 143.546)  1.773 (0.594 – 5.292) | 0.937  0.341  0.020  0.015  0.194  0.851  0.927  0.428  0.685  0.064  0.826  0.622  0.682  0.999  0.927  0.999  0.999  1.000  0.999  0.138  0.305 |
| First MRI (+ vs -)  Cystic  Oedema  Enhancement  Necrosis  Midlineshift | 1.019 (0.210 – 4.942)  0.919 (0.295 – 2.861)  2.412 (0.728 – 7.994)  3.082 (1.036 – 9.169)  2.101 (0.603 – 7.326) | 0.981  0.884  0.150  0.043  0.244 |
|  |  |  |

1. Univariable logistic regression analyses; psychomotor speed

| Baseline variable | Univariable | |
| --- | --- | --- |
|  |  |  |
|  | **OR (95% CI)** | **p-value** |
|  |  |  |
| Age (at time of surgery) | 1.047 (1.008 – 1.088) | 0.017 |
| Gender (male) | 0.336 (0.092 – 1.226) | 0.098 |
| Education  Verhage 6-7 vs lower  Verhage 5-7 vs lower  Verhage 4-7 vs lower  Verhage 3-7 vs lower | 0.409 (0.137 -1.220)  0.493 (0.174 – 1.396)  0.434 (0.078 – 2.419)  0.083 (0.007 -0.970) | 0.109  0.183  0.341  0.047 |
| ASA-score  >2 vs lager  >1 vs lager | 0.000 (0.000 – 0.000)  2.135 (0.638 – 7.141) | 0.999  0.218 |
| Tumor grade (HGG vs LGG) | 3.907 (1.244 – 12.268) | 0.020 |
| Histology  Astrocytoma  Oligodendroglioma  Mixed glioma  Glioblastoma  Ganglioglioma | 0.134 (0.017 – 1.051)  3.000 (0.509 – 17.671)  0.336 (0.092 – 1.226)  3.792 (1.369 – 10.504)  0.000 (0.000 – 0.000) | 0.056  0.225  0.098  0.010  1.000 |
| IDH1 (Mutant vs WT) | 0.227 (0.072 – 0.714) | 0.011 |
| 1p19q deletion (+ vs absent) | 0.686 (0.104 – 4.514) | 0.695 |
| WHO 2016 (+ vs -)  Gr. II/III IDH-M. 1p19q (-)  Gr. II/III IDH-M. 1p19q (+)  Gr. II/III IDH-WT. 1p19q (-)  Gr. IV IDH-M  Gr. IV IDH-WT | 0.164 (0.021 – 1.289)  0.531 (0.143 – 1.970)  0.980 (0.111 – 8.660)  0.000 (0.000 – 0.000)  4.511 (1.597 – 12.738) | 0.086  0.344  0.986  0.999  0.004 |
| Volume pre-operatief (T2 FLAIR) | 1.001 (0.994 – 1.009) | 0.761 |
| Tumor location (involvement + vs -)  Left hemisphere  Right hemisphere  Both hemispheres  Left frontal  Left parietal  Left temporal  Left occipital  Left insula  Left hippocampus  Left thalamus  Right frontal  Right parietal  Right temporal  Right occipital  Right insula  Right hippocampus  Right thalamus  Multifocal  Cortical involvement  Brainstem  Sulcus Centralis | 2.105 (0.572 – 7.752)  0.549 (0.148 – 2.030)  0.000 (0.000 – 0.000)  1.299 (0.484 – 3.487)  1.269 0.440 – 3.662)  1.231 (0.456 – 3.322)  1.219 (0.314 – 4.727)  0.748 (0.273 – 2.052)  1.380 (0.408 – 4.671)  7.143 (1.833 – 27.828)  0.340 (0.074 – 1.568)  0.431 (0.053 – 3.521)  1.118 (0.225 – 5.555)  0.000 (0.000 – 0.000)  0.765 (0.159 – 3.674)  0.000 (0.000 – 0.000)  0.000 (0.000 – 0.000)  0.000 (0.000 – 0.000)  0.713 (0.075 – 6.750)  0.000 (0.000 – 0.000)  1.319 (0.496 – 3.512) | 0.263  0.368  0.999  0.604  0.659  0.682  0.775  0.573  0.604  0.005  0.167  0.432  0.892  0.999  0.738  0.999  0.999  0.999  0.768  0.999  0.579 |
| First MRI (+ vs -)  Cystic  Oedema  Enhancement  Necrosis  Midlineshift | 0.291 (0.036 – 2.327)  1.826 (0.680 – 4.900)  5.752 (1.582 – 20.908)  3.419 (1.253 – 9.325)  0.616 (0.130 – 2.914) | 0.291  0.232  0.008  0.016  0.541 |
|  |  |  |

1. Univariable logistic regression analyses; visuospatial functioning

| Baseline variable | Univariable | |
| --- | --- | --- |
|  |  |  |
|  | **OR (95% CI)** | **p-value** |
|  |  |  |
| Age (at time of surgery) | 1.010 (0.978 – 1.042) | 0.557 |
| Gender (male) | 0.553 (0.187 – 1.632) | 0.283 |
| Education  Verhage 6-7 vs lower  Verhage 5-7 vs lower  Verhage 4-7 vs lower  Verhage 3-7 vs lower | 0.996 (0.979 – 1.014)  0.876 (0.307 – 2.504)  0.995 (0.966 – 1.026)  0.462 (0.040 – 5.332) | 0.689  0.805  0.759  0.536 |
| ASA-score  >2 vs lager  >1 vs lager | 0.000 (0.000 – 0.000)  0.498 (0.173 – 1.435) | 1.000  0.197 |
| Tumor grade (HGG vs LGG) | 0.682 (0.274 – 1.693) | 0.409 |
| Histology  Astrocytoma  Oligodendroglioma  Mixed glioma  Glioblastoma  Ganglioglioma | 1.896 (0.704 – 5.107)  2.275 (0.389 – 13.290)  0.553 (0.187 – 1.632)  0.800 (0.297 – 2.151)  0.000 (0.000 – 0.000) | 0.206  0.361  0.283  0.658  1.000 |
| IDH1 (Mutant vs WT) | 0.684 (0.253 – 1.851) | 0.455 |
| 1p19q deletion (+ vs absent) | 0.430 (0.098 – 1.892) | 0.264 |
| WHO 2016 (+ vs -)  Gr. II/III IDH-M. 1p19q (-)  Gr. II/III IDH-M. 1p19q (+)  Gr. II/III IDH-WT. 1p19q (-)  Gr. IV IDH-M  Gr. IV IDH-WT | 1.480 (0.510 – 4.293)  0.769 (0.257 – 2.299)  6.741 (1.388 – 32.727)  1.071 (0.114 – 10.086)  0.613 (0.189 – 1.988) | 0.470  0.639  0.018  0.952  0.415 |
| Volume pre-operatief (T2 FLAIR) | 0.997 (0.989 – 1.005) | 0.455 |
| Tumor location (involvement + vs -)  Left hemisphere  Right hemisphere  Both hemispheres  Left frontal  Left parietal  Left temporal  Left occipital  Left insula  Left hippocampus  Left thalamus  Right frontal  Right parietal  Right temporal  Right occipital  Right insula  Right hippocampus  Right thalamus  Multifocal  Cortical involvement  Brainstem  Sulcus Centralis | 0.527 (0.196 – 1.421)  2.286 (0.838 – 6.236)  0.000 (0.000 – 0.000)  0.266 (0.095 – 0.740)  0.769 (0.257 – 2.299)  1.540 (0.602 – 3.944)  1.191 (0.303 – 4.691)  0.553 (0.207 – 1.482)  1.083 (0.323 – 3.631)  1.075 (0.212 – 5.454)  2.256 (0.827 – 5.156)  2.389 (0.649 – 8.795)  0.450 (0.054 – 3.749)  0.000 (0.000 – 0.000)  0.571 (0.120 – 2.721)  0.000 (0.000 – 0.000)  1.444 (0.143 – 14.587)  0.000 (0.000 – 0.000)  0.217 (0.029 – 1.637)  4.429 (0.266 – 73.703)  0.907 (0.357 – 2.304) | 0.206  0.106  0.999  0.011  0.639  0.368  0.802  0.239  0.897  0.930  0.112  0.190  0.460  1.000  0.482  0.999  0.755  1.000  0.138  0.300  0.907 |
| First MRI (+ vs -)  Cystic  Oedema  Enhancement  Necrosis  Midlineshift | 0.631 (0.132 – 3.022)  0.765 (0.285 – 2.055)  0.633 (0.249 – 1.607)  0.459 (0.143 – 1.471)  0.000 (0.000 – 0.000) | 0.564  0.595  0.336  0.190  0.998 |
|  |  |  |

1. Univariable logistic regression analyses; memory

| Baseline variable | Univariable | |
| --- | --- | --- |
|  |  |  |
|  | **OR (95% CI)** | **p-value** |
|  |  |  |
| Age (at time of surgery) | 1.024 (0.928 – 1.130) | 0.638 |
| Gender (male) | 0.000 (0.000 – 0.000) | 0.998 |
| Education  Verhage 6-7 vs lower  Verhage 5-7 vs lower  Verhage 4-7 vs lower  Verhage 3-7 vs lower | 0.996 (0.921 – 1.077)  0.996 (0.932 – 1.065)  0.996 (0.920 – 1.078)  0.996 (0.918 – 1.081) | 0.921  0.914  0.921  0.923 |
| ASA-score  >2 vs lager  >1 vs lager | 0.000 (0.000 – 0.000)  0.629 (0.038 – 10.350) | 0.999  0.746 |
| Tumor grade (HGG vs LGG) | 0.675 (0.043 – 10.513) | 0.779 |
| Histology  Astrocytoma  Oligodendroglioma  Mixed glioma  Glioblastoma  Ganglioglioma | 0.000 (0.000 – 0.000)  0.000 (0.000 – 0.000)  2.237 (0.136 – 36.713)  1.562 (0.095 – 25.576)  0.000 (0.000 – 0.000) | 0.998  0.999  0.573  0.754  1.000 |
| IDH1 (Mutant vs WT) | 0.705 (0.043 – 11.582) | 0.807 |
| 1p19q deletion (+ vs absent) | 0.000 (0.000 – 0.000) | 0.998 |
| WHO 2016 (+ vs -)  Gr. II/III IDH-M. 1p19q (-)  Gr. II/III IDH-M. 1p19q (+)  Gr. II/III IDH-WT. 1p19q (-)  Gr. IV IDH-M  Gr. IV IDH-WT | 3.880 (0.234 – 64.218)  0.000 (0.000 – 0.000)  0.000 (0.000 – 0.000)  0.000 (0.000 – 0.000)  2.486 (0.151 – 40.854) | 0.344  0.998  0.999  0.999  0.524 |
| Volume pre-operatief (T2 FLAIR) | 0.965 (0.899 – 1.036) | 0.323 |
| Tumor location (involvement + vs -)  Left hemisphere  Right hemisphere  Both hemispheres  Left frontal  Left parietal  Left temporal  Left occipital  Left insula  Left hippocampus  Left thalamus  Right frontal  Right parietal  Right temporal  Right occipital  Right insula  Right hippocampus  Right thalamus  Multifocal  Cortical involvement  Brainstem  Sulcus Centralis | 0.352 (0.021 – 5.788)  3.241 (0.197 – 53.458)  0.000 (0.000 – 0.000)  0.906 (0.055 – 14.821)  0.000 (0.000 – 0.000)  1.773 (0.108 – 29.045)  0.000 (0.000 – 0.000)  1.440 (0.088 – 23.567)  0.000 (0.000 – 0.000)  0.000 (0.000 – 0.000)  3.207 (0.194 – 52.894)  0.000 (0.000 – 0.000)  0.000 (0.000 – 0.000)  0.000 (0.000 – 0.000)  0.000 (0.000 – 0.000)  0.000 (0.000 – 0.000)  0.000 (0.000 – 0.000)  0.000 (0.000 – 0.000)  0.000 (0.000 – 0.000)  0.000 (0.000 – 0.000)  0.000 (0.000 – 0.000) | 0.465  0.411  0.999  0.945  0.998  0.688  0.999  0.798  0.998  0.999  0.415  0.999  0.999  0.999  0.999  0.999  0.999  1.000  0.999  1.000  0.997 |
| First MRI (+ vs -)  Cystic  Oedema  Enhancement  Necrosis  Midlineshift | 0.000 (0.000 – 0.000)  0.000 (0.000 – 0.000)  0.757 (0.046 – 12.385)  0.000 (0.000 – 0.000)  0.000 (0.000 – 0.000) | 0.999  0.998  0.845  0.998  0.998 |
|  |  |  |

1. Univariable logistic regression analyses; language

| Baseline variable | Univariable | |
| --- | --- | --- |
|  |  |  |
|  | **OR (95% CI)** | **p-value** |
|  |  |  |
| Age (at time of surgery) | 1.039 (0.990 – 1.091) | 0.121 |
| Gender (male) | 0.894 (0.218 – 3.666) | 0.876 |
| Education  Verhage 6-7 vs lower  Verhage 5-7 vs lower  Verhage 4-7 vs lower  Verhage 3-7 vs lower | 0.855 (0.228 – 3.209)  0.768 (0.185 – 3.187)  0.158 (0.025 – 1.001)  0.996 (0.972 – 1.021) | 0.817  0.716  0.050  0.764 |
| ASA-score  >2 vs lager  >1 vs lager | 0.000 (0.000 – 0.000)  2.377 (0.467 – 12.103) | 0.999  0.297 |
| Tumor grade (HGG vs LGG) | 4.570 (0.893 – 23.399) | 0.068 |
| Histology  Astrocytoma  Oligodendroglioma  Mixed glioma  Glioblastoma  Ganglioglioma | 0.303 (0.037 – 2.495)  0.000 (0.000 – 0.000)  0.000 (0.000 – 0.000)  17.270 (2.107 – 141.577)  0.000 (0.000 – 0.000) | 0.267  0.999  0.998  0.008  1.000 |
| IDH1 (Mutant vs WT) | 0.071 (0.009 – 0.594) | 0.015 |
| 1p19q deletion (+ vs absent) | 0.000 (0.000 – 0.000) | 0.999 |
| WHO 2016 (+ vs -)  Gr. II/III IDH-M. 1p19q (-)  Gr. II/III IDH-M. 1p19q (+)  Gr. II/III IDH-WT. 1p19q (-)  Gr. IV IDH-M  Gr. IV IDH-WT | 0.000 (0.000 – 0.000)  0.313 (0.038 – 2.587)  0.000 (0.000 – 0.000)  0.000 (0.000 – 0.000)  12.462 (2.488 – 62.420) | 0.998  0.281  0.999  0.999  0.002 |
| Volume pre-operatief (T2 FLAIR) | 1.004 (0.994 – 1.014) | 0.414 |
| Tumor location (involvement + vs -)  Left hemisphere  Right hemisphere  Both hemispheres  Left frontal  Left parietal  Left temporal  Left occipital  Left insula  Left hippocampus  Left thalamus  Right frontal  Right parietal  Right temporal  Right occipital  Right insula  Right hippocampus  Right thalamus  Multifocal  Cortical involvement  Brainstem  Sulcus Centralis | 1.538 (0.309 – 7.663)  0.750 (0.150 – 3.750)  0.000 (0.000 – 0.000)  0.716 (0.182 – 2.811)  0.743 (0.146 – 3.780)  7.618 (1.503 – 38.612)  0.775 (0.090 – 6.648)  1.818 (0.462 – 7.153)  3.100 (0.824 – 13.746)  4.857 (0.824 – 28.633)  0.357 (0.043 – 2.984)  2.286 (0.425 – 12.291)  2.519 (0.465 – 13.663)  6.625 (0.541 - 81.172)  1.771 (0.336 – 9.348)  0.000 (0.000 – 0.000)  0.000 (0.000 – 0.000)  0.000 (0.000 – 0.000)  0.151 (0.012 – 1.849)  0.000 (0.000 – 0.000)  1.250 (0.318 – 4.908) | 0.599  0.726  0.999  0.632  0.720  0.014  0.816  0.392  0.137  0.081  0.342  0.335  0.284  0.139  0.500  0.999  0.999  1.000  0.139  1.000  0.749 |
| First MRI (+ vs -)  Cystic  Oedema  Enhancement  Necrosis  Midlineshift | 0.689 (0.081 – 5.836)  1.769 (0.482 – 6.494)  0.000 (0.000 – 0.000)  6.356 (1.540 – 26.241)  3.833 (0.972 – 15.116) | 0.732  0.390  0.997  0.011  0.055 |
|  |  |  |
