# Supplementary material for: Neurocognitive changes after awake surgery in glioma patients: a retrospective cohort study
Source: J Neurooncol. 2019 Dec 4;146(1):97–109. doi: 10.1007/s11060-019-03341-6 (PMC6938472; doi:10.1007/s11060-019-03341-6)
Supplement: Supplementary file 5 — Electronic supplementary material 5: Online Resource Table 1 (DOCX 21 kb) Multivariable linear regression analyses for predicting delta-Z-scores [file 11060_2019_3341_MOESM5_ESM.docx]

| Online Resource table 1: Baseline characteristics |  | Mean (min-max); Frequency (%)* |
| --- | --- | --- |
| Age (at time of surgery; in years) |  | 51.69 (19 - 82) |
| Gender (male) |  | 116 (69.0 %) |
| Level of education (Verhage classification)  *1*  *2*  *3*  *4*  *5*  *6*  *7* |  | 0 (0.0 %)  4 (2.4 %)  8 (4.9 %)  37 (22.6 %)  44 (26.8 %)  36 (22.0 %)  35 (21.3 %) |
| ASA-score**  ASA 1  ASA 2  ASA 3 |  | 52 (36.4 %)  87 (60.8 %)  4 (2.8 %) |
| Histology  Astrocytoma  Oligodendroglioma  Oligoastrocytoma  Glioblastoma  Ganglioglioma |  | 39 (23.2 %)  8 (4.8 %)  43 (25.6 %)  76 (45.2 %)  2 (1.2 %) |
| Tumor grade  WHO II  WHO III  WHO IV |  | 64 (38.1 %)  28 (16.7 %)  76 (45.2 %) |
| IDH1 (mutant) |  | 75 (48.3 %) |
| WHO 2016 classification  Gr II/III IDH-M 1p19q del (-)  Gr II/III IDH-M 1p19q del (+)  Gr II/III IDH-WT 1p19q del (-)  Gr IV IDH-M  Gr IV IDH-WT |  | 32 (22.9 %)  35 (25.0 %)  9 (6.4 %)  7 (5.0 %)  57 (40.7 %) |
| Tumor volume (cm^3^) |  | 69.10 (2.79-277.78) |
| Tumor location T2 (FLAIR)  Left  Right  Both sided  Left frontal (+)  Left parietal (+)  Left temporal (+)  Left occipital (+)  Left insula (+)  Left hippocampus (+)  Left thalamus (+)  Right frontal (+)  Right parietal (+)  Right temporal (+)  Right occipital (+)  Right insula (+)  Right hippocampus (+)  Right thalamus (+) |  | 118 (70.2 %)  44 (26.2 %)  6 (3.6 %)  85 (51.2 %)  42 (25.3 %)  61 (36.7 %)  20 (11.9 %)  68 (41.0 %)  30 (18.1 %)  12 (7.2 %)  43 (25.9 %)  24 (14.5 %)  20 (11.9 %)  5 (3.0 %)  28 (16.9 %)  3 (1.8 %)  5 (3.0 %) |
| Treatment after surgery  None  Only RT  Stupp  Only temozolomide  RT+PCV  PCV  RT+temozolomide |  | 50 (29.8 %)  25 (14.9 %)  66 (39.3 %)  6 (3.6 %)  15 (8.9 %)  1 (0.6 %)  5 (3.0 %) |
| Loss to follow-up |  | 34 (20.2 %) |

**Percentages do not add up to 100% for certain variables due to missing values

**Although the Karnofsky Performance Score (KPS) is more commonly used to indicate the functional impairments of glioma patients, we also collected ASA-scores to measure physical status since NCF problems may influence KPS, but are less likely to determine ASA-score.
