# Supplementary material for: Neurocognitive changes after awake surgery in glioma patients: a retrospective cohort study
Source: J Neurooncol. 2019 Dec 4;146(1):97–109. doi: 10.1007/s11060-019-03341-6 (PMC6938472; doi:10.1007/s11060-019-03341-6)
Supplement: Supplementary file 6 — Electronic supplementary material 6: Online Resource Table 2 (DOCX 17 kb) Neuropsychological tasks per domain [file 11060_2019_3341_MOESM6_ESM.docx]

| Online Resource table 2: Neuropsychological tasks per domain |
| --- |
| Attention & Executive Functioning |
| Wechsler Adult Intelligence Scale (WAIS) Digit Span Forward^1^  Trail Making Test (TMT) Switching ratio (TMTB/TMTA)^2^  Phonologic fluency^3^  Stroop/Delis Kaplan Executive Function System (DKEFS) inhibition ratio^4^ |
| Memory |
| WAIS Digit Span Backward  15 Words test (WT) direct, delay, recognition^5^  Rey-Osterieth Complex Figure Test (ROCF) delay^6^  Semantic Fluency^7^ |
| Language |
| Boston Naming Test^8^  Token Test^9^ |
| Visuospatial functioning |
| Judgment of Line Orientation (JULO)^10^  ROCF direct |
| Psychomotor Speed |
| Stroop/DKEFS I  Stroop/DKEFS II  TMTA |

1. Wechsler Adult Intelligence Scale Third Edition Digit Span [WAIS-III] (WAIS-III Administration and scoring manual, 1997), Wechsler Adult Intelligence Scale Fourth Edition Digit Span [WAIS-IV] (WAIS-IV-NL Technische handleiding, 2013)
2. Trail Making Test [TMT] (Giovagnoli, Del Pesce, Mascheroni, Simoncelli, Laiacona, & Capitani, 1996)
3. Phonologic Verbal Fluency Test [Lexical Fluency] (Harrison, Buxton, Husain, & Wise, 2010; Schmand, Groenink, & Van Den Dungen, 2008)
4. The Stroop Color and Word Test [Stroop] (MacLeod, 1991), Color Word Interference Test (Benton, Sivan, Hamsher, Varney, & Spreen, 1994)
5. 15 Words Test [15WT] (Saan & Deelman, 1986)
6. Rey-Osterieth Complex Figure Test [ROCF] (Berry & Carpenter, 1992; Spreen & Strauss, 1998)
7. Semantic Verbal Fluency Test [Semantic Fluency] (Harrison et al., 2010)
8. Boston Naming Task [BNT] (Heesbeen & Van Loon-Vervoorn, 2001)
9. Token Test [TT] (Boller & Vignolo, 1966)
10. Judgment of Line Orientation [JULO] (Benton, Sivan, Hamsher, Varney, & Spreen, 1994; Benton, Varney, & Hamsher, 1978)
